# The Role of Total Quality Management in the Pharmaceutical, Food, and Nutritional Supplement Sectors

**DOI:** 10.3390/foods13162606

**Published:** 2024-08-20

**Authors:** Vassilios Vassos, Agathi Voltezou, Agathangelos Stavropoulos, Elisavet Stavropoulou, Christos Stefanis, Christina Tsigalou, Evangelia Nena, Ekaterini Chatzaki, Theodoros C. Constantinidis, Eugenia Bezirtzoglou

**Affiliations:** 1 Laboratory of Hygiene and Environmental Protection, Medical School, Democritus University of Thrace, 68100 Alexandroupolis, Greece; vasilhsvassos@yahoo.gr (V.V.); lvoltez@admin.duth.gr (A.V.); elisabeth.stavropoulou@gmail.com (E.S.); ctsigalo@med.duth.gr (C.T.); tconstan@med.duth.gr (T.C.C.); empezirt@med.duth.gr (E.B.); 2 School of Social and Political Sciences, University of Glasgow, Glasgow G12 8RT, UK; angelostavrop@gmail.com; 3 Laboratory of Microbiology, National Kapodistrian University of Athens, 11527 Athens, Greece; 4 Laboratory of Social Medicine, Medical School, Democritus University of Thrace, 68100 Alexandroupolis, Greece; enena@med.duth.gr; 5 Laboratory of Pharmacology, Medical School, Democritus University of Thrace, 68100 Alexandroupolis, Greece; achatzak@med.duth.gr

**Keywords:** Total Quality Management, TQM, public health, pharmaceutical, food, nutritional supplement, sustainability

## Abstract

Total Quality Management (TQM) is a holistic approach widely adopted across industries to ensure quality control and management. This document examines TQM practices in the pharmaceutical, food, and nutritional supplement sectors, highlighting their vital role in public health, sustainability, and consumer acceptance. By analyzing the literature and case studies, the article demonstrates how TQM significantly ensures product safety and quality. Real-world examples and empirical evidence showcase the benefits of TQM methodologies, from rigorous quality control to efficient management processes, helping to meet and exceed regulatory standards. The article also underscores TQM’s critical role in addressing sustainability challenges, integrating eco-friendly practices, reducing waste, and optimizing resources. Furthermore, TQM fosters consumer trust and loyalty through transparency, continuous improvement, and responsiveness to feedback, building lasting business–customer relationships. In conclusion, this manuscript illuminates TQM’s multifaceted impact on the pharmaceutical, food, and nutritional supplement sectors, presenting it as a pivotal framework for safeguarding public health, promoting sustainability, and enhancing consumer acceptance in a dynamic global landscape.

## 1. Introduction

### Overview of Total Quality Management (TQM) and Its Importance

Effective TQM practices significantly protect and promote public health [1,2]. TQM is pivotal in safeguarding public health by preventing defects, maintaining product safety, and ensuring compliance with regulatory requirements [3]. Real-world case studies and data analysis provide concrete examples of TQM’s positive impact on public health [4].

Total Quality Management (TQM) has evolved into a foundational quality control and management framework across diverse industries [5]. In this opinion paper, we explore the application of TQM principles in the pharmaceutical, food, and nutritional supplement industries [6]. Specifically, we examine how TQM practices contribute to public health protection, sustainability initiatives, and consumer acceptance [7].

Total Quality Management (TQM) practices are closely aligned with regulatory compliance in the pharmaceutical [8], food [9], and nutritional supplement industries. Total Quality Management (TQM) seamlessly incorporates sustainability and environmental management, embodying an innovative approach to foster cooperative sustainability goals. This strategy sheds light on novel economic construction and social life perspectives, exemplified by the pandemic, to better navigate and mitigate its adverse effects [9]. Regulatory bodies, such as the U.S. Food and Drug Administration (FDA) and the European Medicines Agency (EMA), have established strict guidelines and standards for product quality and safety. TQM provides a systematic approach to ensure these regulations are consistently met.

Public health is a critical concern [2], especially in the pharmaceutical industry, where the quality and safety of medicines directly impact patient well-being. In the pharmaceutical sector, regulatory compliance is non-negotiable [10]. Deviations from Good Manufacturing Practices (GMPs) [11], International Organization for Standardization ISO 9000 standards, and other quality standards can result in severe consequences, including product recalls and legal actions. By embracing TQM principles, pharmaceutical companies maintain compliance and exceed the regulatory requirements in pursuit of excellence in ensuring the production of safe and effective medications [12]. These practices have been instrumental in reducing medication errors, ensuring accurate labeling, and preventing contamination [13]. TQM practices focus on quality control and assurance, process optimization, and adherence to regulatory requirements [13], thereby mitigating risks to public health. 

In the food industry [9], TQM is pivotal in ensuring food safety and preventing foodborne illnesses. TQM helps manufacturers meet stringent regulations related to food safety [14]. The Food Safety Modernization Act (FSMA) in the United States [15] has set new food production and distribution standards. TQM, mainly through Hazard Analysis and Critical Control Points (HACCP), provides a systematic framework for adhering to these regulations [16]. Hazard Analysis and Critical Control Points (HACCP) are TQM principles that guide identifying and controlling potential hazards throughout food production [17]. The food industry can protect consumers from health risks associated with contaminated or unsafe products by adhering to these principles. It aids in identifying potential hazards, implementing controls, and maintaining detailed records to demonstrate compliance [18].

A surge in worldwide supplement sales can be primarily attributed to the heightened enthusiasm for supplements as potential preventive measures against CoronaVIrus Disease of 2019 (COVID-19) by supporting or enhancing immune function [19]. TQM principles extend to the nutritional supplement industry [20], where product quality and safety are paramount.

These principles ensure that nutritional supplements meet established standards and do not pose health risks to consumers. TQM practices enhance consumer confidence in the nutritional supplement market through quality control, transparency, and adherence to regulatory requirements [21]. However, regulatory requirements may vary from country to country. TQM practices are vital in ensuring that products meet these diverse standards. By adhering to a comprehensive quality management system, manufacturers can navigate the complex landscape of international regulations, ultimately certifying the safety and quality of their products [22].

Total Quality Management (TQM) is a holistic approach to long-term success that warrants stable progress in all aspects of an organization as a process and not as a short-term goal. Moreover, it increases product quality and services through ongoing refinements in response to continuous feedback. Each industry applies TQM differently, depending on its unique standards, challenges, and goals.

Total Quality Management (TQM) is a thorough and widely accepted methodology for quality control and management on a global scale. This study explores the intricate implementation of TQM practices, focusing specifically on the pharmaceutical, food, and nutritional supplement sectors. It underlines these practices’ critical role in ensuring public health, advocating for sustainability, and improving consumer acceptance.

Through meticulous exploration, the article highlights how TQM practices significantly contribute to ensuring the safety and quality of products in these crucial industries. Beyond quality control, TQM provides a structured framework for integrating eco-friendly practices into production processes, reducing waste, and optimizing resource utilization. The ecological considerations emphasized in this study showcase TQM as a catalyst for responsible and sustainable business practices. This study is primarily addressed to a broad audience that includes the following addressees:

Academics and researchers: Those involved in public health, pharmaceutical studies, food safety, and nutritional sciences. The detailed exploration of TQM practices provides a valuable reference for ongoing research and academic discourse.

Industry professionals: Managers and quality assurance professionals in the pharmaceutical, food, and nutritional supplement sectors. The practical examples and case studies illustrate the implementation of TQM principles and their benefits.

Regulatory bodies: Organizations responsible for documenting and enforcing quality standards. The discussion on regulatory compliance and the pivotal role of TQM in meeting these standards offers insights into effective policy-making. 

Consumers and public health advocates: individuals interested in understanding how TQM impacts product safety, quality, and sustainability in industries critical to public health.

Furthermore, the article underlines how TQM is a cornerstone in fostering consumer trust and loyalty. Companies in these sectors can establish and maintain credibility by aligning with TQM principles, ultimately strengthening consumer relationships. This manuscript aims to explore the implementation and impact of Total Quality Management (TQM) practices across the pharmaceutical, food, and nutritional supplement sectors. The specific purpose is to highlight how TQM secures compliance with regulatory standards and contributes to broader public health, sustainability, and consumer trust. The article addresses the gap in the existing literature by providing a detailed analysis of TQM’s multifaceted role in these industries, particularly in improving product safety and quality. It transcends the notion of TQM as a mere set of methodologies, portraying it as a linchpin for safeguarding public health, promoting sustainability, and enhancing consumer acceptance in the dynamic global landscape.

To address the overarching themes of TQM’s application and its impact, this study seeks to answer the following research questions:

How do TQM practices contribute to public health protection and consumer trust in the pharmaceutical, food, and nutritional supplement sectors?

What TQM methodologies are employed in the pharmaceutical, food, and nutritional supplement industries, and how do they ensure regulatory compliance and sustainability?

What are the challenges and future directions in implementing TQM in the pharmaceutical, food, and nutritional supplement sectors?

## 2. Materials and Methods

### 2.1. Literature Review Process

The methodology employed in this document involves thoroughly examining the existing literature and comprehensive case studies to elucidate the application and impact of Total Quality Management (TQM) practices in the pharmaceutical, food, and nutritional supplement sectors [16,17]. The initial step includes a literature review, encompassing scholarly articles, industry reports, and relevant publications to gather insights into TQM’s theoretical foundations and practical implementations. Subsequently, a detailed analysis of case studies from these industries is conducted, providing real-world examples that illustrate the effectiveness of TQM methodologies [17]. The synthesis of information from both the literature and case studies allows for a nuanced exploration of the multifaceted contributions of TQM in ensuring product safety, regulatory compliance, sustainability, and consumer acceptance. This methodology aims to provide a comprehensive and evidence-based understanding of the role of TQM in these critical sectors, drawing upon a diverse range of sources to support the manuscript’s insights and conclusions.

### 2.2. Bibliometric Analysis

**Figure 1:**
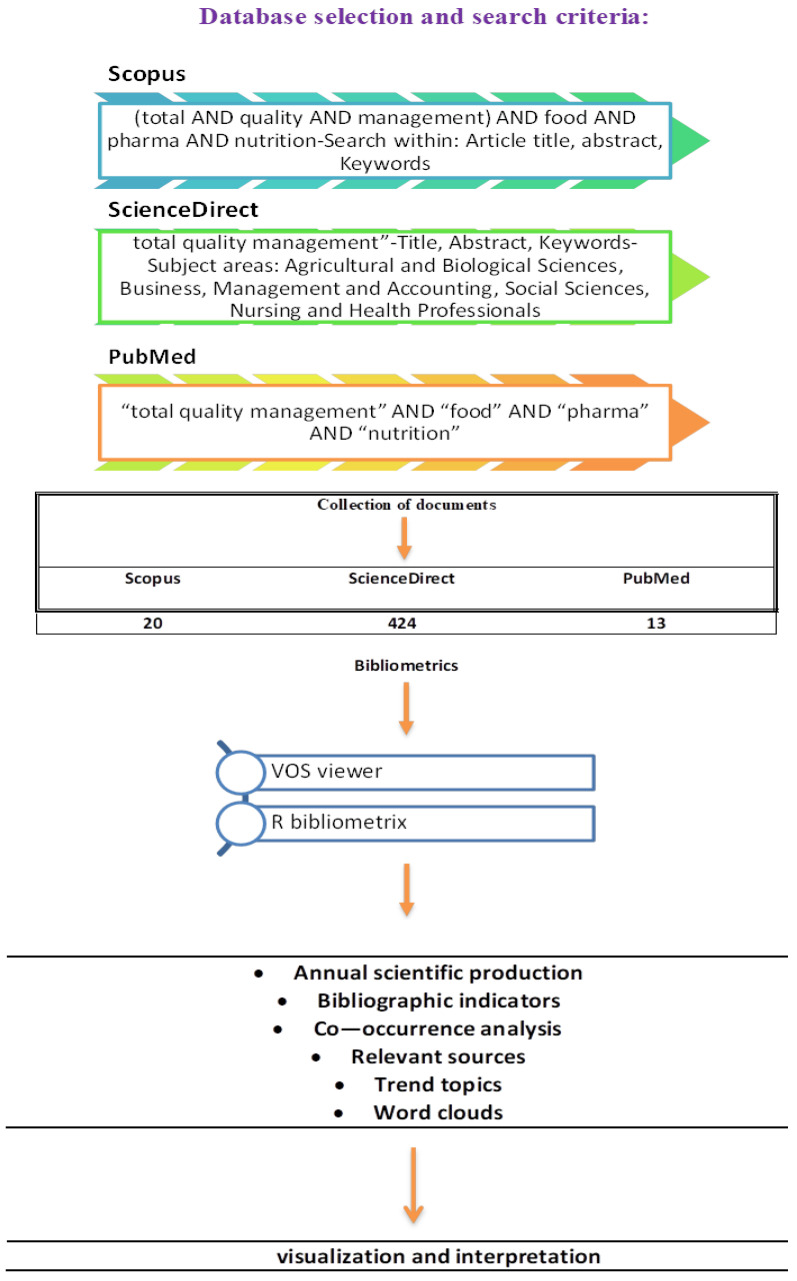
Research flow diagram.

Beyond the literature review, we conducted a bibliometric analysis to spotlight research trends and illuminate the scientific landscape of total quality management and the research progress made in the last decade. The research involved searching the Scopus, ScienceDirect, and PubMed databases. Boolean Syntax was a powerful tool for combining keywords with various Boolean operators, crucial for efficient document retrieval. Scopus, for example, was mainly chosen due to its extensive coverage, including 1.7 billion cited references and nearly 2500 serial titles from approximately 7000 publishers across top-level subject fields such as life, social, physical, and health sciences. The Scopus database offers significant advantages, including diversification, flexibility in research fields, and an advanced document analyzer mechanism based on Boolean Syntax, facilitating sophisticated indexing procedures and distributing numerous scientific journals. After thorough experimentation with different keyword combinations, we selected “total quality management”, “food”, “pharma”, and “nutrition”. The search was conducted from the earliest available date in all databases up to 30 April 2024, with the language set to English. The search parameters and the research flow are depicted in Figure 1, ensuring the comprehensiveness of our study [23,24,25].

Figure 1 illustrates the process of selecting databases, formulating search criteria, collecting documents, and using bibliometric tools to visualize and interpret the data. The databases searched include Scopus, ScienceDirect, and PubMed, with specific search terms and subject areas targeted. The collected documents are then analyzed using bibliometric tools to visualize and interpret various bibliographic indicators and trends.

This search encompassed documents such as research articles, reviews, mini-reviews, and manuscripts from conference proceedings and reviews. The documents were collected in a Microsoft Excel sheet, categorized by publication year, subject area, document type, and authors’ institutional affiliations. We utilized advanced tools like Visualization of Similarities Viewer (VOS) Viewer version 1.6.20 and R bibliometrix software 4.4.0 for visualization and bibliographic mapping of the results. Co-occurrence analysis was applied to investigate keyword occurrences within the manuscripts’ titles, abstracts, and texts. The bibliometric analysis followed a structured approach, establishing research criteria and questions and analyzing parameters such as year and subject area. This process culminated in creating networks and visual figures and interpreting these outcomes [23,24,25].

## 3. Results and Discussion

**Figure 2:**
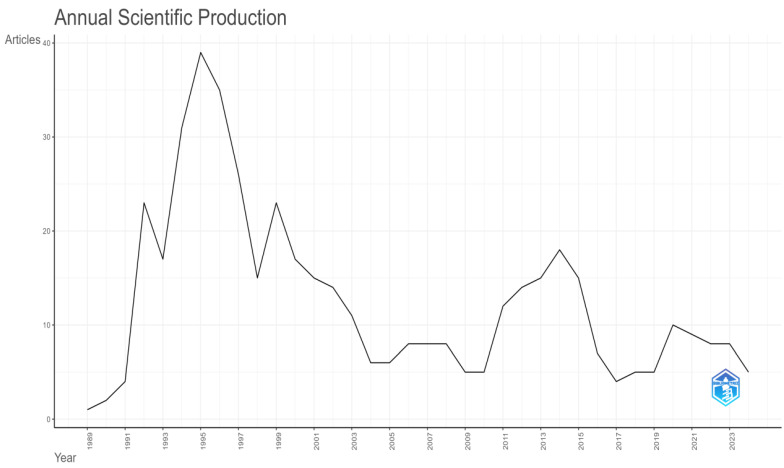
Annual scientific production.

Figure 2, “Annual scientific production”, shows the yearly number of scientific articles published from 1989 to 2023. The *y*-axis indicates the number of articles (ranging from 0 to 40), and the *x*-axis displays the years.

The trend began with only a few publications in 1989 and then saw a rapid increase, with the highest point reached around 1998, including almost 40 articles. There was a noticeable decrease after this peak, hitting a low point in the early 2000s. Another peak emerged around 2010, although it was lower than the previous one. After this peak, the number of publications gradually decreased, with minor fluctuations, resulting in fewer published articles in recent years until 2023. The data show significant variability in scientific production over the analyzed period, with marked peaks and declines indicating periods of higher and lower research activity. The total number of documents collected was 457, as illustrated in the research flow diagram (Figure 2).

**Figure 3:**
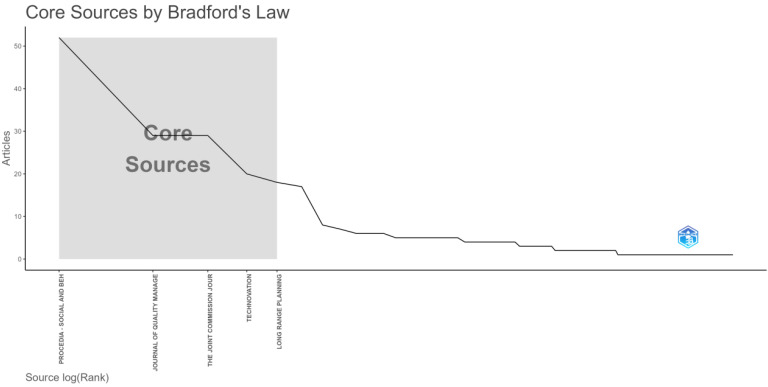
Core sources by Bradford’s law.

Figure 3 depicts the distribution of articles across various sources, identifying the core sources of scientific literature. The *y*-axis represents the number of articles, ranging from 0 to 50, while the *x*-axis represents the sources ranked logarithmically.

The data are plotted as a line graph, starting at a high point with the source *Procedia—Social and Behavioral Sciences* contributing the most articles, over 50. As the ranking progresses, the number of articles per source declines sharply, transitioning into a steady decline. The first few sources are highlighted as the “Core Sources”, contributing a substantial portion of the total articles.

Notable findings reveal that the initial sources, particularly *Procedia—Social and Behavioral Sciences* and *Journal of Quality Management*, significantly outpace others regarding article contributions. Following these core sources, there is a distinct drop in the number of articles from each subsequent source. The tail end of the graph displays a lengthy list of sources, each making a minor contribution, thereby illustrating Bradford’s law. This law suggests that relatively few journals publish most articles on a given topic. This visualization effectively underscores the prominence of the initial sources, thereby highlighting the concentration of knowledge within a few key journals.

**Figure 4:**
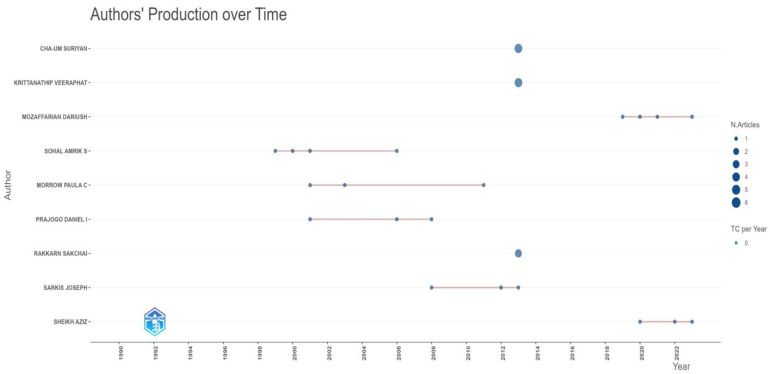
Authors’ production over time.

Figure 4 provides a visual summary of authors’ publication timelines and productivity, highlighting their activity periods and the intensity of their contributions regarding the number of articles published. The horizontal axis represents the timeline from 1990 to 2024, with intervals marked every two years, indicating when the authors published their work, while the vertical axis lists the names of various authors, each reflecting their respective publication activity over time. Each dot along the timeline corresponds to an article published by the author in that particular year, with the dots varying to represent the number of articles published, as indicated by the legend—the larger the dot, the more articles the author published that year. Thin horizontal lines connecting some dots suggest periods of continuous publication activity, revealing varying degrees of productivity among the authors. The figure reveals varying degrees of publication activity among the authors; for instance, Sohal Amrik S has multiple publications connected by lines, indicating continuous output over consecutive years, while authors like Sheikh Aziz have fewer publications, with activity spread across a wide range of years, from the 1990s to the 2010s and 2020s. For example, Sohal Amrik S, Morrow Paula C, Prajogo Daniel I, and Sarkis Joseph demonstrate sustained productivity with multiple publications over several years, as indicated by connected dots on the timeline, highlighting their active years in scholarly publishing. In contrast, authors like Cha-Um Suriyan, Krittanathip Veerahat, Mozaffarian Dariush, Rakkarn Sakchal, and Sheilh Aziz show a limited number of publications, often restricted to one or two entries, with significant gaps or isolated publication years.

**Figure 5:**
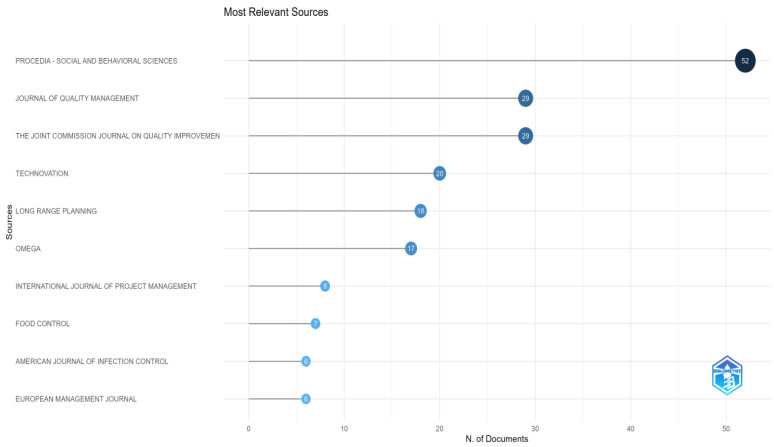
Most relevant sources.

The figure titled “Most relevant sources” ranks the sources based on the number of documents they have published. The *y*-axis lists the sources, while the *x*-axis represents the number of documents published (Figure 5).

Key elements of the visualization include dots representing each source, with their position along the *x*-axis indicating the number of documents they have published. The number within each dot represents the count of documents, providing a clear visual representation of the publication frequency across different sources.

Notably, the top contributors, such as *Procedia—Social and Behavioral Sciences*, with 52 published documents, have significantly impacted the field. Their high publication frequency underscores their role as significant outlets for articles. Similarly, *Journal of Quality Management* and *The Joint Commission Journal on Quality Improvement* have 29 documents each, and they have emerged as important sources of quality management and improvement-related research.

Other notable sources include *Technovation*, with 20 documents, highlighting its focus on innovation and technology management, and *Long Range Planning*, with 18 documents, emphasizing its relevance in strategic and long-term planning research. *Omega* has contributed 17 documents showcasing its operational research and management science involvement.

It is important to note that even sources with fewer publications, such as the *International Journal of Project Management*, *Food Control*, *American Journal of Infection Control*, and *European Management Journal* (each with six to eight documents), play a crucial role in their respective areas. They disseminate significant research findings, contributing to the overall knowledge base of the research community.

This visualization effectively highlights the most relevant sources in document publication, showcasing the journals and sources significantly contributing to the literature. It provides a comprehensive overview of the distribution of scholarly output, illustrating the varying contribution levels across different journals and helping to identify critical sources within the research community.

**Figure 6:**
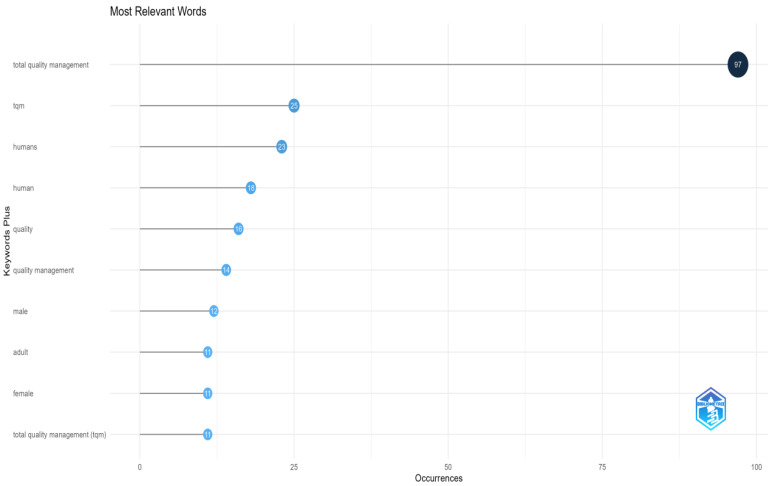
Most relevant words—Keywords Plus.

The figure titled “Most relevant words—Keywords Plus” displays the most frequently occurring keywords in the dataset. The *y*-axis lists the keywords, while the *x*-axis represents the number of occurrences (Figure 6). Key elements of the visualization include dots that correspond to each keyword, with their position along the *x*-axis indicating the number of times the keyword appears. The number within each dot represents the count of occurrences, clearly representing the Keywords Plus within the dataset.

Notably, the keyword “Total quality management” is the most frequent, appearing 97 times. This high frequency underscores its central importance and dominance among the research themes. Following this, “TQM” appears 25 times, highlighting its relevance as an abbreviated form of the primary keyword. Other frequently appearing keywords include “humans” with 23 occurrences and “human” with 18 occurrences, indicating a significant focus on human-related studies. Additionally, “quality” appears 16 times, and “quality management” appears 14 times, emphasizing the importance of quality and its management within the research. The diversity of the research areas reflected in the keywords is worth noting. For instance, “male” and “female” appear 11 times, “adult” appears 11 times, and “total quality management (tqm)” appears 11 times. These keywords reflect diverse focus areas within the research, including demographic factors and specific terms related to quality management. This visualization effectively highlights the key topics and focus areas within the dataset, showcasing the most relevant keywords and providing insight into the primary themes of the research. By illustrating the frequency of these keywords, the visualization helps to identify the dominant areas of interest and the focus of the scholarly community, offering a comprehensive overview of the research landscape.

**Figure 7:**
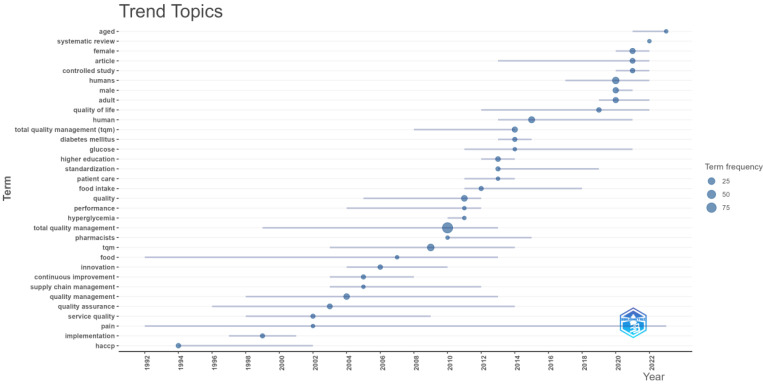
Trend topics in Keywords Plus.

The visualization in Figure 7 effectively captures the temporal trends of critical terms in Keywords Plus, offering insights into the shifting focus and emerging themes in the literature over the analyzed period.

“Trend topics” is a crucial tool in our analysis, as it visualizes the frequency of various terms over time from 1992 to 2023. The *y*-axis lists the terms, while the *x*-axis represents the years. The size of the dots reveals the frequency of term occurrences, with larger dots representing higher frequencies. This comprehensive view of the evolving scientific literature is essential for understanding the context of our analysis.

The visualization’s key elements include dots representing the occurrence of a term in a specific year. The size of each dot corresponds to the frequency of the term in that year, making it easy to identify which terms were most prevalent during specific periods.

Our observations reveal that “Total quality management”, “TQM”, “quality management”, “quality”, and “continuous improvement” have appeared consistently over the years, indicating sustained importance in the scientific literature. Terms like “aged”, “systematic review”, “female”, “controlled study”, “humans”, “male”, and “quality of life” have shown increasing relevance in recent years, particularly from 2010 onwards, highlighting their growing significance. This underlines the relevance and impact of our research.

Keywords such as “higher education”, “innovation”, “performance”, “service quality”, and “implementation” also demonstrate notable occurrences over time, suggesting their essential roles in ongoing research. Additionally, keywords such as “diabetes mellitus”, “glucose”, “hyperglycemia”, “patient care”, “food intake”, and “pharmacists” appear more frequently in recent years, indicating emerging focus areas in these fields. The visual distribution of dots shows the evolving trends and highlights the terms that have gained prominence over different periods. This visualization provides a clear overview of the trends in critical topics over the analyzed period, showcasing the shifting focus and emerging themes within the scientific literature. It effectively illustrates how specific topics have maintained their importance while new areas of interest have emerged, reflecting the dynamic nature of scientific research.

**Figure 8:**
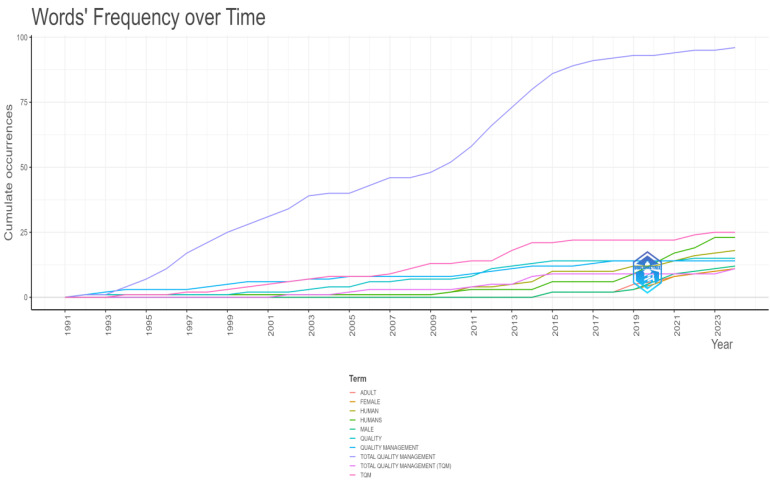
Words’ frequency over time—Keywords Plus.

Figure 8 discloses the cumulative occurrences of selected keywords from 1991 to 2024 of Keywords Plus. The *y*-axis represents the cumulative occurrences, while the *x*-axis represents the years.

The critical elements of the visualization are designed for clarity and ease of understanding. Lines representing the cumulative frequency of specific terms over time are differentiated by colors, enabling straightforward identification of trends and patterns.

Observations reveal that “Quality” (represented by the cyan line) has the highest cumulative occurrences, steadily increasing over the years and surpassing 100 occurrences by 2023. “Total quality management” (pink line) and “TQM” (purple line) also show significant upward trends, with “Total quality management” displaying notable increases around 2020.

Terms like “Quality management” (green line), “Humans” (dark green line), “Human” (light green line), “Male” (blue line), and “Female” (orange line) are not just showing gradual increases in cumulative occurrences. However, they also indicate their relevance and sustained importance in the dataset. “Adult” (red line) also shows growth, though slower than the top terms, reflecting its ongoing significance but with a less pronounced increase. This visualization serves as a comprehensive guide to the evolution of key terms over time, effectively showcasing the growing emphasis and sustained importance of certain concepts within the scientific literature. By illustrating the cumulative occurrences of these terms, the visualization offers a detailed overview of how key topics have evolved, highlighting longstanding focus areas and emerging trends within the research community (Figure 7).

**Figure 9:**
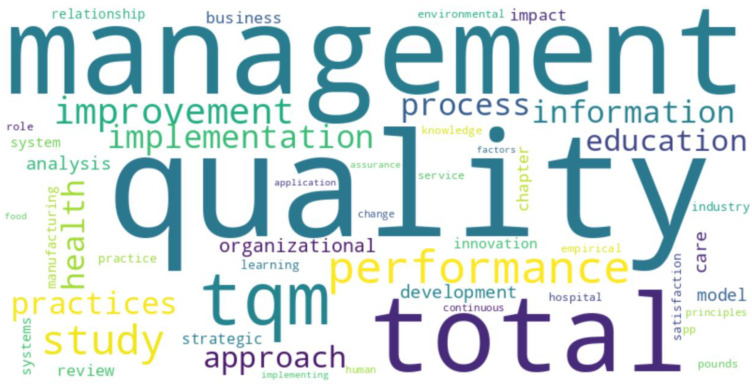
Word cloud of top 50 words in titles.

Figure 9, “Top 50 words in titles”, presents a word cloud that visualizes the frequency of the 50 most common terms in manuscript titles. In this visualization, the size of each word is proportional to its frequency of occurrence, with more significant words appearing more frequently in the dataset.

**Figure 10:**
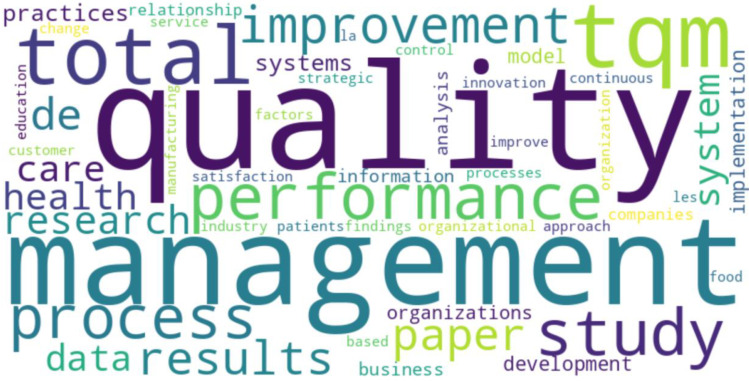
Word cloud of top 50 words in abstracts.

Figure 10 discloses the Top 50 words in abstracts, illustrating the frequency of the 50 most common terms. In this visualization, the size of each word is proportional to its frequency of occurrence, with more prominent words appearing more frequently in the dataset.

Dominant terms such as “quality”, “management”, and “total” are not just the most prominent, but they signify the backbone of the research, indicating their significant presence in the abstracts and suggesting that they are central themes within the body of literature. Other significant words like “improvement”, “performance”, “health”, “system”, “process”, and “research” also appear prominently, highlighting critical areas of focus in the research. Furthermore, the inclusion of diverse topics such as “health”, “care”, “innovation”, and “practices” not only showcases the interdisciplinary nature of the research but also underlines the breadth of the research. Quality management and performance improvement principles are not confined to a single field. However, they are being applied across different fields, including healthcare, education, and industrial practices, thereby broadening the scope and impact of the research.

The word cloud includes a variety of terms spanning different aspects of research and industry. Words like “care”, “data”, “results”, “information”, “study”, and “implementation” indicate a broad spectrum of research interests, from patient care and data analysis to the implementation of new practices and the reporting of research findings. More specific terms such as “TQM”, “practices”, “organizations”, “innovation”, and “continuous” show the depth and specificity of the research topics covered, suggesting that the articles delve into detailed and specialized discussions within broader themes.

**Figure 11:**
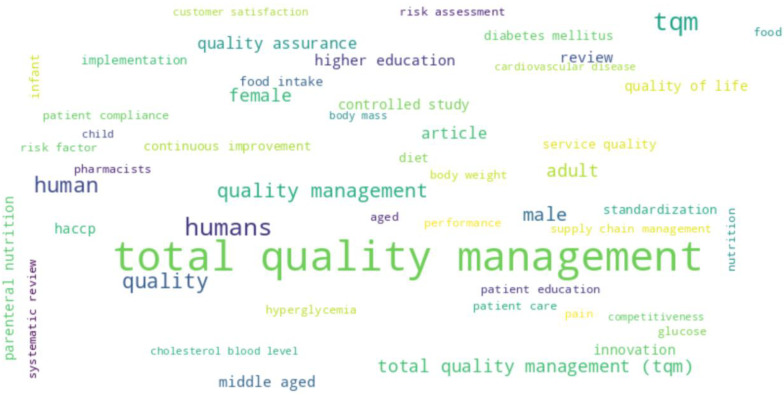
Word cloud of top 50 words in authors’ keywords.

The figure titled “Top 50 words in authors’ keywords” presents a word cloud that visualizes the frequency of the 50 most common terms found in the keywords provided by authors in their documents. In this visualization, the size of each word is proportional to its frequency of occurrence, with more prominent words appearing more frequently in the dataset (Figure 11).

The word cloud prominently features vital terms such as “total quality management”, “quality management”, and “tqm”. These terms, which are the most dominant in the cloud, underscore their significant presence in the authors’ keywords. This suggests that the central themes within the body of literature revolve around concepts related to quality management.

Other significant words like “quality”, “humans”, “human”, “adult”, and “female” are also prominently featured, indicating critical areas of focus in the research. These terms suggest that researchers frequently explore a wide range of topics, including human studies, demographic factors, and quality aspects, thereby highlighting the multidisciplinary nature of the research.

The presence of terms such as “quality assurance”, “service quality”, and “continuous improvement” indicates an ongoing interest in enhancing efficiency and effectiveness within various sectors. Furthermore, the inclusion of diverse topics such as “health”, “education”, “innovation”, and “patient care” showcases the interdisciplinary nature of the research. Quality management and performance improvement principles are applied across different fields, including healthcare, education, and industrial practices.

More specialized terms like “systematic review”, “haccp”, and “hyperglycemia” point to a detailed exploration of these themes, emphasizing the structured and systemic approach taken by researchers. These terms suggest a focus on strategic planning, organizational dynamics, and system-level analyses, which are crucial for understanding and improving complex processes and structures.

**Figure 12:**
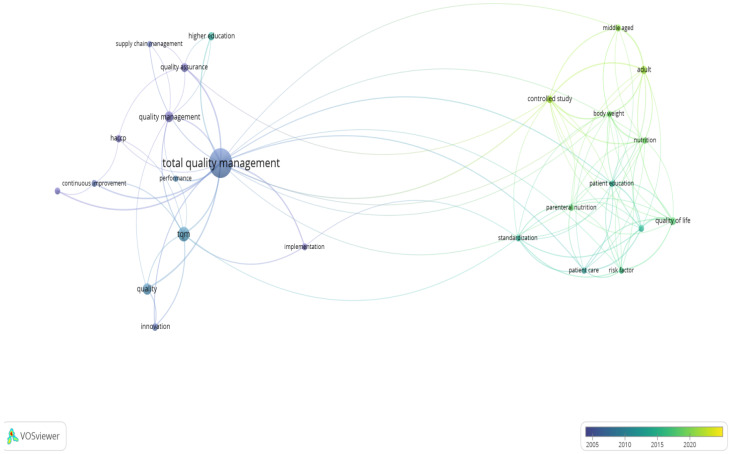
Evolution of the research on Total Quality Management.

The “Timeline visualization of keywords in Total Quality Management” figure visualizes the evolution and interconnections of keywords related to Total Quality Management (TQM) over time. This network map was created using VOSviewer 1.6.20, with a timeline from 2004 to 2024 indicated by the color gradient at the bottom (Figure 12).

The size of each node (dot) indicates the frequency of keyword occurrences, with larger nodes representing higher frequencies. The color gradient from dark to light blue represents the progression of time, with darker shades indicating earlier years and lighter shades indicating more recent years. Key elements of the visualization include the network of lines connecting different keywords, illustrating how these terms co-occur within the research articles. The central keyword, “total quality management”, is not just prominently displayed but stands out, indicating its significant presence and centrality in the network. This term is connected and linked to other keywords, suggesting its broad applicability and relevance across multiple research areas, as it has consistently appeared over the years.

Other significant keywords, such as “quality management”, “TQM”, “quality”, “innovation”, and “continuous improvement” also appear prominently, highlighting their sustained importance in the literature. The centrality and connectivity of these keywords suggest their potential as key research areas, with the ability to influence and be influenced by a wide range of other topics.

Terms such as “quality assurance”, “implementation”, “performance”, “higher education”, and “supply chain management” demonstrate notable occurrences, suggesting their essential roles in the research landscape. The visualization shows not just an increase but a surge in relevance for keywords like “quality of life”, “controlled study”, “nutrition”, “patient care”, and “standardization”, particularly in more recent years, highlighting the emergence of exciting new areas of focus within the scientific literature. The visual distribution of nodes and connections shows the evolving trends and highlights the terms that have gained prominence over different periods. However, it is essential to note that this visualization is based on a specific set of research articles and may only partially represent part of the scientific literature in the field. Therefore, the trends and prominence of terms should be interpreted with this limitation in mind. This visualization reveals how specific topics have maintained their importance while new areas of interest have emerged, reflecting the dynamic nature of scientific research.

Table 1 presents a comprehensive list of ISO standards relevant to the food, pharmaceutical, and nutritional supplement industries. Each section lists ISO standards that outline specific, detailed requirements, guidelines, or methodologies. The respective standards and procedures are designed to safeguard these sectors’ quality, safety, and compliance.

A systematic review combined with bibliometric techniques of research trends and frontiers is a reliable method of recording the current state of research in a domain. However, limitations still emerge in these studies as well. The subjective interpretation of the results of various case studies and the impossibility of searching all the bibliographic databases introduce the first limitations of any research study. Specifically, the comparison, implementation, and effectiveness of Total Quality Management (TQM) practices in different geographic areas and industries, even in companies of various sizes, cannot be universally captured in just one survey. Direct surveys or experiments, which could offer additional empirical evidence, are also absent. Finally, contemporary perspectives and new research topics, such as sustainability and consumer perceptions of specific food categories [26], are constantly emerging. Although such issues are manifested in this document, they could also be independent topics to investigate in future research studies.

## 4. Challenges and Future Directions

Total Quality Management (TQM) plays a vital role in the pharmaceutical, food, and nutritional supplement sectors by directly impacting product quality, safety, and regulatory compliance. In the pharmaceutical industry, TQM practices are vital for ensuring that medications are produced consistently, safely, and effectively, minimizing contamination risk and ensuring adherence to Good Manufacturing Practices (GMPs). These practices streamline processes, reduce errors, and enhance the overall reliability of pharmaceutical products, which is critical for patient safety. Similarly, in the food industry, TQM helps manufacturers comply with stringent food safety regulations, such as the HACCP system (Hazard Analysis and Critical Control Points), by identifying potential hazards, implementing preventive measures, and maintaining detailed records to ensure food products are safe for consumption. This approach not only safeguards public health but also builds consumer trust [27].

In the nutritional supplements industry, TQM is essential for maintaining quality standards and ensuring products are free from harmful substances. Industry professionals use TQM to manage the complexity of sourcing diverse ingredients, maintaining consistency in product formulations, and meeting regulatory requirements across various markets. By systematically applying TQM practices, professionals in these sectors can reduce the risk of defects, contamination, and non-compliance, improving product quality, enhancing consumer satisfaction, and maintaining compliance with evolving regulatory demands. Additionally, TQM fosters continuous improvement, innovation, and sustainability in their operations, ultimately leading to greater market competitiveness and strengthened consumer trust.

While TQM practices have significantly contributed to public health, sustainability, and consumer acceptance in the pharmaceutical, food, and nutritional supplement industries, challenges remain [28]. In the pharmaceutical sector, there is a constant need to balance innovation with safety and quality. Ensuring the safety of new drugs and therapies while expediting their development is an ongoing challenge [28]. The food industry faces challenges related to supply chain complexity and global sourcing. Ensuring that products meet safety standards, regardless of origin, remains a significant challenge [29]. Additionally, as consumer preferences evolve, companies must stay agile in responding to changing trends while maintaining safety and quality.

In the nutritional supplement industry, the challenge lies in maintaining product consistency and preventing contamination, given the variety of ingredients used. Regulatory harmonization across regions also presents a challenge, as companies often need to navigate differing standards in different markets [30].

Looking to the future, TQM practices will continue to evolve. Integrating digital technologies, such as blockchain for supply chain transparency and advanced analytics for quality control, will be vital to maintaining and improving TQM systems. Sustainability practices are expected to become even more prominent, with companies striving to minimize their environmental footprint and support ethical sourcing [31].

Pharmaceutical, food, and nutritional manufacturers are leveraging digitalization to enhance product traceability. In the pharmaceutical industry, digitalization has been a game-changer in various aspects, including research and development, manufacturing, and distribution [32]. For example, adopting electronic batch records and real-time monitoring systems has significantly improved pharmaceutical products’ quality control and traceability [33].

TQM principles are deeply ingrained in the pharmaceutical sector, strongly emphasizing Good Manufacturing Practices (GMPs). By integrating digitalization with TQM, pharmaceutical companies have enhanced product quality, streamlined regulatory compliance, and improved supply chain transparency [34].

One of the critical areas where digitalization and TQM have made a substantial impact is ensuring the authenticity and safety of pharmaceutical products. Counterfeit drugs pose a significant threat to public health, and stringent measures are required to combat this problem. Through the use of track-and-trace technologies and digital authentication systems [34], pharmaceutical companies can guarantee the integrity of their products [35]. This safeguards public health and builds trust with healthcare professionals and consumers. Additionally, digitalization has played a vital role in pharmacovigilance, the practice of monitoring the safety of drugs post-market [36]. Adverse events and side effects can be reported and analyzed in real-time, enabling swift responses and necessary product recalls if safety concerns arise.

Digitalization and TQM practices are transforming the landscape of food safety and quality assurance in the food industry. Food processing companies are adopting cutting-edge technologies to monitor and control various aspects of their operations [37,38]. One of the most critical areas of concern in the food industry is contamination and foodborne illnesses [39,40]. To address this issue, digitalization monitors and controls factors such as temperature, humidity, and cleanliness throughout the food production and supply chain. IoT (Internet of Things) devices provide real-time data on these variables, ensuring food products meet safety and quality standards [41].

Furthermore, TQM principles are applied in the food industry by implementing stringent quality control measures at various stages of production. Employees are trained to adhere to these quality standards, and continuous improvement is encouraged. The synergy with digitalization [42] ensures that data from quality control checks are immediately accessible and can be analyzed to identify trends or anomalies. This promotes a culture of quality and accountability [43]. The nutritional supplement industry has been under increased scrutiny recently due to product authenticity and safety concerns. Consumers often rely on these products to improve their health and well-being, making it essential to uphold quality standards [44].

## 5. Conclusions

Total Quality Management (TQM) practices safeguard public health, promote sustainability, and enhance consumer acceptance in the pharmaceutical, food, and nutritional supplement industries. These sectors have achieved significant progress by emphasizing quality control, regulatory compliance, continuous improvement, innovation, supplier development, and consumer education.

The adherence to stringent quality control measures and sustainability initiatives has profound implications for public health protection and environmental conservation. Consumer trust and acceptance are closely linked to the transparency, safety, and consistency promoted by TQM.

Moreover, TQM positions the customer at the center of quality improvement efforts, encompassing end consumers, healthcare professionals, regulatory agencies, and other stakeholders. Meeting and exceeding customer expectations is vital for building trust and ensuring long-term success.

Despite existing challenges, continuous innovation and sustainability efforts are not only important but vital for the future of TQM. TQM remains a cornerstone of excellence in these industries, supporting public health and consumer satisfaction.

The integration of digitalization with TQM principles is pivotal in enhancing public health, advancing sustainability, and elevating consumer acceptance. Digital technologies provide real-time data, improve regulatory compliance, and enhance traceability, allowing organizations to address quality and safety concerns proactively. Digitalization promotes transparency and reduces environmental footprints, while TQM fosters a commitment to quality, continuous improvement, and customer-centric approaches.

In conclusion, the synergy between digitalization and TQM in the pharmaceutical, food, and nutritional supplement industries ensures product safety, quality, authenticity, and sustainability.

## Figures and Tables

**Table 1:** Overview of ISO standards in the food, pharmaceutical, and nutritional supplement industries.

ISO Standards— Food Industry ^1,2^	Title
ISO 22000	Food safety management systems—Requirements for any organization in the food chain
ISO 22002-1	Prerequisite programme on food safety—Part 1: Food manufacturing
ISO 22003	Food safety management systems—Requirements for bodies providing audit and certification
ISO 22004	Food safety management systems—Guidance on the application of ISO 22000
ISO 22005	Traceability in the feed and food chain—General principles and basic requirements for system design and implementation
ISO 6887	Microbiology of the Food Chain Package Parts 1 to 4: ISO 6887-1, ISO 6887-2, ISO 6887-3 and ISO 6887-4
ISO/TS 22002	Academy Food Collection Prerequisite Programs on Food Safety—ISO/TS 22002-1, ISO/TS 22002-2, ISO/TS 22002-3 and ISO/TS 22002-4
ISO 22000:2018	Food Safety Management Systems—A Practical Guide
ISO 22000, ISO/TS 22003, ISO 22004 and ISO 22005	Food Safety Package
ISO 22000:2018 ISO 22000:2018	Food safety management systems—Requirements for any organization in the food chain
ISO 22174:2005	Microbiology of food and animal feeding stuffs—Polymerase chain reaction (PCR) for the detection of food-borne pathogens—General requirements and definitions
ISO 19020:2017	Microbiology of the food chain—Horizontal method for the immunoenzymatic detection of staphylococcal enterotoxins in foodstuffs
ISO 26642:2010	Food products—Determination of the glycaemic index (GI) and recommendation for food classification
ISO 22003-1:2022	Food safety—Part 1: Requirements for bodies providing audit and certification of food safety management systems
ISO/TS 17728:2015	Microbiology of the food chain—Sampling techniques for microbiological analysis of food and feed samples
ISO/TS 22002-1:2009	Prerequisite programmes on food safety—Part 1: Food manufacturing
ISO/TS 22002-4:2013	Prerequisite programmes on food safety—Part 4: Food packaging manufacturing
ISO/TS 22002-6:2016	Prerequisite programmes on food safety—Part 6: Feed and animal food production
**ISO Standards—** **Pharmaceutical Industry**	**Title**
ISO 15378	Primary packaging materials for medicinal products—Particular requirements for the application of ISO 9001:2008, with reference to Good Manufacturing Practice (GMP)
ISO 9001	Quality management systems—Requirements
ISO 13485	Medical devices—Quality management systems—Requirements for regulatory purposes
ISO 14644-1	Cleanrooms and associated controlled environments—Part 1: Classification of air cleanliness by particle concentration
ISO 14375:2018	Child-resistant non-reclosable packaging for pharmaceutical products—Requirements and testing
ISO 22413:2021	Transfer sets for pharmaceutical preparations—Requirements and test methods
ISO 28862:2018	Packaging—Child-resistant packaging—Requirements and testing procedures for non-reclosable packages for non-pharmaceutical products
ISO 8871-1:2003	Elastomeric parts for parenterals and for devices for pharmaceutical use—Part 1: Extractables in aqueous autoclavates
ISO 8871-2:2020	Elastomeric parts for parenterals and for devices for pharmaceutical use—Part 2: Identification and characterization
ISO 8871-5:2016	Elastomeric parts for parenterals and for devices for pharmaceutical use—Part 5: Functional requirements and testing
ISO 11418-1:2016	Containers and accessories for pharmaceutical preparations—Part 1: Drop-dispensing glass bottles
ISO/HL7 27953-1:2011	Health informatics—Individual case safety reports (ICSRs) in pharmacovigilance—Part 1: Framework for adverse event reporting
ISO/HL7 27953-2:2011	Health informatics—Individual case safety reports (ICSRs) in pharmacovigilance—Part 2: Human pharmaceutical reporting requirements for ICSR
**ISO Standards—** **Nutritional Supplement** **Industry**	**Title**
ISO 22000	Food safety management systems—Requirements for any organization in the food chain
ISO 9001	Quality management systems—Requirements
ISO 22716	Cosmetics — Good Manufacturing Practices (GMP)—Guidelines on Good Manufacturing Practices
ISO 22579:2020	Infant formula and adult nutritionals—Determination of fructans—High performance anion exchange chromatography with pulsed amperometric detection (HPAEC-PAD) after enzymatic treatment
ISO 20636:2018	Infant formula and adult nutritionals—Determination of vitamin D by liquid chromatography-mass spectrometry
ISO 20637:2015	Infant formula and adult nutritionals—Determination of myo-inositol by liquid chromatography and pulsed amperometry
ISO 20647:2015	Infant formula and adult nutritionals—Determination of total iodine—Inductively coupled plasma mass spectrometry (ICP-MS)
ISO 21422:2018	Milk, milk products, infant formula and adult nutritionals—Determination of chloride—Potentiometric titration method
ISO 4214:2022	Milk and milk products—Determination of amino acids in infant and adult/paediatric nutritional formulas and dairy products
ISO 15151:2018	Milk, milk products, infant formula and adult nutritionals—Determination of minerals and trace elements—Inductively coupled plasma atomic emission spectrometry (ICP-AES) method
ISO 16958:2015	Milk, milk products, infant formula and adult nutritionals—Determination of fatty acids composition—Capillary gas chromatographic method
ISO 20633:2015	Infant formula and adult nutritionals—Determination of vitamin E and vitamin A by normal phase high performance liquid chromatography
ISO 20634:2015	Infant formula and adult nutritionals—Determination of vitamin B12 by reversed phase high performance liquid chromatography (RP-HPLC)
ISO 20635:2018	Infant formula and adult nutritionals—Determination of vitamin C by (ultra) high performance liquid chromatography with ultraviolet detection ((U)HPLC-UV)
ISO 20639:2015	Infant formula and adult nutritionals—Determination of pantothenic acid by ultra high performance liquid chromatography and tandem mass spectrometry method (UHPLC-MS/MS)
ISO 20649:2015	Infant formula and adult nutritionals—Determination of chromium, selenium and molybdenum—Inductively coupled plasma mass spectrometry (ICP-MS)
ISO 21424:2018	Milk, milk products, infant formula and adult nutritionals—Determination of minerals and trace elements—Inductively coupled plasma mass spectrometry (ICP-MS) method
ISO 21446:2019	Infant formula and adult nutritionals—Determination of trans and total (cis + trans) vitamin K1 content—Normal phase HPLC
ISO 21468:2020	Infant formula and adult nutritionals—Determination of free and total choline and free and total carnitine—Liquid chromatography tandem mass spectrometry (HPLC-MS/MS)
ISO 21470:2020	Infant formula and adult nutritionals—Simultaneous determination of total vitamins B1, B2, B3 and B6—Enzymatic digestion and LC-MS/MS
ISO 23305:2020	Fortified milk powders, infant formula and adult nutritionals—Determination of total biotin by liquid chromatography coupled with immunoaffinity column clean-up extraction
ISO 23443:2020	Infant formula and adult nutritionals—Determination of ?-carotene, lycopene and lutein by reversed-phase ultra-high performance liquid chromatography (RP-UHPLC)
ISO 20784:2021	Sensory analysis—Guidance on substantiation for sensory and consumer product claims

^1^ https://www.iso.org/search.html?PROD_isoorg_en%5Bquery%5D=food%20safety&PROD_isoorg_en%5Bmenu%5D%5Bfacet%5D=standard (Last accessed: 7 May 2024). ^2^. https://webstore.ansi.org/search/find?st=food&v=5&cp=1&f1=Standard&f1=Package&f2=2&f3=39 (Last accessed: 7 May 2024).

## Data Availability

No new data were created or analyzed in this study. Data sharing is not applicable to this article.
